# The Prognostic Value and Immune Landscapes of a m^6^A/m^5^C/m^1^A-Related LncRNAs Signature in Head and Neck Squamous Cell Carcinoma

**DOI:** 10.3389/fcell.2021.718974

**Published:** 2021-11-30

**Authors:** Enhao Wang, Yang Li, Ruijie Ming, Jiahui Wei, Peiyu Du, Peng Zhou, Shimin Zong, Hongjun Xiao

**Affiliations:** ^1^ Department of Otorhinolaryngology, Union Hospital, Tongji Medical College, Huazhong University of Science and Technology, Wuhan, China; ^2^ Department of Stomatology, Zhongshan Hospital, Fudan University, Shanghai, China

**Keywords:** RNA methylation, long non-coding RNA, prognostic signature, head and neck squamous cell carcinoma, epigenetic change

## Abstract

**Background:** N6-methyladenosine (m^6^A), 5-methylcytosine (m^5^C) and N1-methyladenosine (m^1^A) are the main RNA methylation modifications involved in the progression of cancer. However, it is still unclear whether m^6^A/m^5^C/m^1^A-related long non-coding RNAs (lncRNAs) affect the prognosis of head and neck squamous cell carcinoma (HNSCC).

**Methods:** We summarized 52 m^6^A/m^5^C/m^1^A-related genes, downloaded 44 normal samples and 501 HNSCC tumor samples with RNA-seq data and clinical information from The Cancer Genome Atlas (TCGA) database, and then searched for m^6^A/m^5^C/m^1^A-related genes co-expressed lncRNAs. We adopt the least absolute shrinkage and selection operator (LASSO) Cox regression to obtain m^6^A/m^5^C/m^1^A-related lncRNAs to construct a prognostic signature of HNSCC.

**Results:** This prognostic signature is based on six m^6^A/m^5^C/m^1^A-related lncRNAs (AL035587.1, AC009121.3, AF131215.5, FMR1-IT1, AC106820.5, PTOV1-AS2). It was found that the high-risk subgroup has worse overall survival (OS) than the low-risk subgroup. Moreover, the results showed that most immune checkpoint genes were significantly different between the two risk groups (*p* < 0.05). Immunity microenvironment analysis showed that the contents of NK cell resting, macrophages M2, and neutrophils in samples of low-risk group were significantly lower than those of high-risk group (*p* < 0.05), while the contents of B cells navie, plasma cells, and T cells regulatory (Tregs) were on the contrary (*p* < 0.05). In addition, patients with high tumor mutational burden (TMB) had the worse overall survival than those with low tumor mutational burden.

**Conclusion:** Our study elucidated how m^6^A/m^5^C/m^1^A-related lncRNAs are related to the prognosis, immune microenvironment, and TMB of HNSCC. In the future, these m^6^A/m^5^C/m^1^A-related lncRNAs may become a new choice for immunotherapy of HNSCC.

## 1. Introduction

Head and neck squamous cell carcinoma (HNSCC) represents the sixth most common malignant tumor in the world (Johnson et al., 2020). These tumors originate from the mucosa of various anatomical parts of the oral cavity, pharynx, larynx and sinonasal tract and are heterogeneous in etiology and clinicopathological characteristics, and these highly aggressive malignant tumors affect more than 830,000 patients worldwide each year (Johnson et al., 2020; Qin et al., 2021). HNSCC is usually associated with exposure to tobacco-derived carcinogens, excessive alcohol consumption, or both. Tumors appearing in the oropharynx are increasingly related to human papillomavirus (HPV) carcinogenic strains, mainly HPV-16, and to a lesser extent HPV-18 and other strains of infection (Riva et al., 2021). In current treatment methods, only about 50% of HNSCC patients survive for more than 5 years. Therefore, it is urgent to analyze the heterogeneity of HNSCC to choose different treatment options. In particular, there is an urgent need for reliable biomarkers with predictive and prognostic value (Rasheduzzaman et al., 2020).

Some evidences show that epigenetic changes (including: DNA methylation, histone covalent modification, chromatin remodeling, non-coding RNA (ncRNA) activity and RNA chemical modifications) are often related to oral carcinogenesis, tumor malignancy and resistance to treatment (Castilho et al., 2017; Romanowska et al., 2020). Increasing evidence suggests that dynamic RNA modifications pathways are also misregulated in human cancers (including HNSCC, lung cancer, breast cancer, pancreatic cancer, endometrial cancer, et al.) and may be ideal targets of cancer therapy (Barbieri and Kouzarides, 2020; Zhou et al., 2020; Huang et al., 2021). Nowadays, over 100 types of chemical modifications have been identified in cellular RNAs, including messenger RNAs (mRNAs), transfer RNAs (tRNAs), ribosomal RNAs (rRNAs), and ncRNAs, most of these modifications contribute to pre-mRNA splicing, nuclear exporting, transcript stability and translation initiation of eukaryotic cellular RNAs. Among these modifications, methylation modification is the most abundant which include N6-methyladenosine (m^6^A), 5-methylcytosine (m^5^C), N1-methyladenosine (m^1^A) (Shi et al., 2020). M^6^A is the most abundant form of methylation modification in eukaryotic cellular RNAs, and it is also the most thoroughly studied type of RNA modification. The sequence near the m6A modification site on the mRNA is highly conservative, mainly occurring on the adenine of the RRACH (R: purine; A: m^6^A; H: non-guanine) sequence, and its function is determined by the methyltransferase (writers: methyltransferase like 3 (METTL3), methyltransferase like 14 (METTL14) and WT1 associated protein (WTAP), et al.), demethylase (erasers: fat mass and obesity associated protein (FTO) and alkB homologue 5 (ALKBH5), et al.) and binding protein (readers: YTH domain containing family, the insulin like growth factor 2 mRNA binding protein (IGF2BP) family and heterogeneous nuclear ribonucleoproteins A2/B1 (HNRNPA2B1), et al.) are jointly regulated (Yang et al., 2018; Li M. et al., 2021). M^6^A modification is involved in a variety of biological processes, such as stem cell differentiation, cell division, gametogenesis and biological rhythms, et al., as well as the occurrence of a variety of diseases, including tumors, obesity and infertility (Xu Y. et al., 2020; Zhang M. et al., 2020; Song et al., 2020). M^5^C modification is another prevalent RNA modification in multiple RNA species, including mRNAs, tRNAs, rRNAs, and ncRNAs (Chen et al., 2021). M^5^C modification is distributed with mRNA, enriched around 5′UTR and 3′UTR, and conserved in tRNA and rRNA. It is dynamically regulated by related enzymes, including methyltransferase (writers: NOL1/NOP2/SUN domain family member (NSUN) family and DNA methyltransferase like 2 (DNMT2), et al.), demethylases (erasers: tet methylcytosine dioxygenase 2 (TET2), et al.), and binding proteins (readers: Aly/REF export factor (ALYREF), et al.) (Wiener and Schwartz, 2021). Up to now, accumulated studies have shown that m^5^C is involved in various RNA metabolism, including mRNA export, RNA stability and translation. The absence of m^5^C modification in organisms may lead to mitochondrial dysfunction, stress response defects, gametogenesis and embryogenesis frustration, and abnormal development of nerves and brain, which is related to cell migration and tumorigenesis (Chellamuthu and Gray, 2020; Vissers et al., 2020). The majority of these m^1^A sites are located within the 5′-untranslated region (5′ UTR), m^1^A modification has been identified in tRNA, rRNA, mRNA, long non-coding RNA (lncRNA) and mitochondrial (mt) transcripts, It can also be dynamically regulated by methyltransferases, demethylases and binding proteins (Zhang and Jia, 2018).

LncRNAs are a class of RNA molecules with transcript length exceeding 200nt. it is generally believed that they do not encode proteins, but participate in the regulation of protein-encoding genes in many aspects (epigenetic regulation, transcriptional regulation and post-transcriptional regulation, etc.) in the form of RNA. More and more lncRNAs have been found to play a role in the initiation and development of HNSCC, suggesting that they can be used as novel biomarkers and therapeutic targets to provide more effective diagnosis, prognosis and treatment for HNSCC patients, such as HOTAIR, UCA1, FOXC1, AFAP1-AS1, et al. (Cossu et al., 2019; Wang S. et al., 2020; Wang et al., 2020c.). Researchers have found that methylation modification of lncRNA regulates numerous processes affecting cancer cell activity by enhancing and increasing lncRNA stability, increasing lncRNA nuclear accumulation, and promoting decay of some lncRNAs (Dai et al., 2020). However, there is currently no evidence to explain the correlation between three major RNA methylation modifications related lncRNAs and HNSCC. In this study, we integrated 44 normal samples and 501 tumor samples with clinical trials data from The Cancer Genome Atlas (TCGA). We used bioinformatics and statistical analysis methods to explore whether m^6^A/m^5^C/m^1^A-related lncRNAs were related to the diagnosis and prognosis of HNSCC.

## 2. Materials and Methods

### 2.1. Data Collection and Processing

RNA-seq data and clinical information of HNSCC patients were downloaded from The Cancer Genome Atlas (TCGA) database (https://cancergenome.nih.gov/) (Wang et al., 2020d), which contained data of 501 HNSCC and 44 non-tumor tissues. Raw count data was downloaded for further analysis. In addition, based on previous publications, the expression matrix of 52 m^6^A/m^5^C/m^1^A-related genes was extracted from TCGA database, including expression data on writers, erasers and readers (Table 1). We obtain the gene annotation file of Genome Reference Consortium Human Build 38 (GRCh38) from the GENCODE website (https://www.gencodegenes.org/human/) for the annotation of the mRNAs in the TCGA database (Frankish et al., 2019).

**TABLE 1:** RNA methylation-related genes of m^6^A/m^5^C/m^1^A.

RNA methylayion	Writer	Reader	Eraser
m6A	METTL3, METTL14, METTL16, WTAP, KIAA1499, RBM15, RBM15B, RBM1, ZC3H13	YTHDC1, YTHDC2, YTHDF1, YTHDF2, YTHDF3, IGF2BP1, IGF2BP2, IGF2BP3, HNRNPA2B1, HNRNPC, HNRNPG, RBMX, FMR1, LRPPRC	FTO, ALKBH5
m5C	NOP2, NSUN1, NSUN2, NSUN3, NSUN4, NSUN5, NSUN7, DNMT1, TRDMT1, DNMT3A, DNMT3B	ALYREF	TET2, YBX1
m1A	TRMT6, TRMT61A, TRMT61B, TRMT61C, TRMT10C, BMT2, RRP8	YTHDF1, YTHDF2, YTHDF3, YTHDC1	ALKBH1, ALKBH3

The simple nucleotide variation files with masked somatic mutation data were acquired from the Genomic Data Commons (GDC) Data Portal (https://portal.gdc.cancer.gov/) (Gong et al., 2018).

We downloaded 33 types of tumor HTSeq-FPKM GDC Hub files, Phenotype GDC Hub files, survival data GDC Hub files from the UCSC Xena database (http://xena.ucsc.edu/) (Yang et al., 2019), and downloaded immune subtype files from the TCGA Pan-Cancer (PANCAN) database. We downloaded the nci 60 RNA seq files from Processed Data Set and Compound activity: DTP NCI-60 files from the CellMiner database (http://discover.nci.nih.gov/cellminer/home.do) (Reinhold et al., 2019).

### 2.2. Statistical Analysis and Bioinformatic Analysis

#### 2.2.1. mRNAs–lncRNAs Co-Expression Network and Prognostic Value of m^6^A/m^5^C/m^1^A-Related lncRNAs

After obtaining the expression matrix of 52 m^6^A/m^5^C/m^1^A-related genes and expression matrix of lncRNAs from the TCGA database, we screened out the lncRNAs co-expressed with m^6^A/m^5^C/m^1^A-related genes by setting the absolute value of correlation coefficient > 0.5 and *p* value < 0.001. The univariate cox regression analysis was performed to screen out prognostic value of m^6^A/m^5^C/m^1^A-related lncRNAs.

#### 2.2.2. Cluster the Samples Based on the m^6^A/m^5^C/m^1^A-Related lncRNAs

Based on the expression level of these 44 lncRNAs with prognostic value in tumor samples, we used the R package “ConsensusClusterPlus” to classify tumor samples into different groups and to analyze the prognosis by cluster analysis of samples in the database (Zheng et al., 2020). Principal Component Analysis (PCA) was applied to the typed samples to observe whether the samples could be intuitively distinguished (Rapa et al., 2021). In order to understand the prognosis of samples with different classifications, the R package “survival” and “Survminer” (Zhang L.-P. et al., 2020) were used to analyze the prognosis of the samples after classifications. Clinical data were included to observe whether different clinical features differed in different classifications. The NMT classification comes from the TNM classification of malignant tumours (TNM) (https://www.uicc.org/resources/tnm), which is a globally recognised standard for classifying the extent of spread of cancer. Each individual aspect of TNM is termed as a category: T category describes the primary tumor site and size; N category describes the regional lymph node involvement; M category describes the presence or otherwise of distant metastatic spread. Gene enrichment analysis (GSEA) was used to understand the differentially functional enrichment pathways of samples with different types (Tang et al., 2021). In order to explore the characteristics of immune microenvironment of different types of samples, the R package “limma” was used to filter the differences in immune cell infiltration of different types of samples (Li Z. et al., 2021). In addition, we further analyzed the expression of 29 immune checkpoint moleclues in different types of samples, including CD70, TNFSF9, FGL1, CD276, NT5E, HHLA2, VTCN1, TNFRSF18, CD274, PDCDL1LG2, IDO1, CTLA4, ICOS, HAVCR, PDCD1, LAG3, SIGLEC15, TNFSF4, TNFRSF9, ENTPD1, VSIR, ICOSLG, TNFSF14, TMIGD2, CD27, NCR3, BTLA, CD40LG, CD40, TNFRSF4 (Zhang C. et al., 2020). Moreover, we also analyzed the differences in the expression levels of the tumor suppressor gene TP53 among the subtypes.

#### 2.2.3. A Prognostic Signature Constructed by m^6^A/m^5^C/m^1^A-Related lncRNAs

The least absolute shrinkage and selection operator (LASSO) cox regression was used to select the most significant prognostic lncRNAs in 44 m6A/m5C/m1A-related lncRNAs for predictive model construction (Wang S. et al., 2020). Lasso is a linear regression method based on L1-regularization. L1-regularization can reduce the complexity of the model and the risk of over-fitting of the model. The explanation in regression analysis is that if two characteristic variables are highly correlated, the regression analysis without regularization may give them approximately equal weight, so the weighted result is still small, but due to the large weight, the over-fitting problem is caused. L1-regularization greatly increases the stability of modeling and achieves the purpose of selecting characteristic variables. The most outstanding advantage of Lasso lies in the penalized regression analysis of all variable coefficients, the relatively unimportant independent variable coefficients become 0, which is excluded from modeling (Vaid et al., 2021; Yu et al., 2021; Hoq et al., 2021). However, the 44 lncRNAs are too large for constructing a prognosis model, so we used LASSO regression analysis to further compress the 44 lncRNAs to get the lncRNAs with the most prognostic value, and their correlation coefficients. Combining respective correlation coefficients of obtained m^6^A/m^5^C/m^1^A-related lncRNAs, the respective gene expression level of these lncRNAs in each tumor sample, and the risk score calculating formula (The risk score calculating formula is: $Risk\;score=\overset{n}{\underset{i=1}{\sum }}\;Coefi*xi$ . where Coefi means the coefficients, xi is the FPKM value of each m^6^A/m^5^C/m^1^A-related lncRNAs), we can grade each sample. All samples can be sorted according to their respective scores. At last, with the median of sample scores as the boundary, tumor samples with scores lower than the median of all scores were divided into low risk group, while tumor samples with scores higher than the median of all scores were divided into high risk group. Finally, a prognostic model of risk score was constructed based on these obtained m^6^A/m^5^C/m^1^A-related lncRNAs. The R package “survival” was used to analyze the survival of the high-risk group and low-risk group in HNSCC. Tumor samples were then divided into a training set (249 samples) and a testing set (249 samples), and there was no statistically significant difference in clinical characteristics between the two groups (Supplementary Table S1). The survival conditions of the high-risk group and low-risk group were analyzed in the training set, and the results were verified in the testing set. The univariate and multivariate regressions were further used to analyze whether risk score in the training set and validation set could be used as an independent prognostic indicator. In addition, PCA and T-Distributed Neighbor Embedding (T-SNE) analysis (Wang W. et al., 2021) were used to visualize whether the high-risk group and low-risk group could be distinguished by risk score. Receiver operating characteristic (ROC) curve was used to test model effectiveness (Longlong et al., 2020). Next, we evaluated the clinical characteristics of the high-risk group and the low-risk group: age, gender, HPV-status, tumor stage, tumor grade, and tumor TNM, and performed Gene Ontology (GO) and Kyoto Encyclopedia of Genes and Genomes (KEGG) analysis of the genes that differed between the two groups (Wu et al., 2021).

Considering the strength expression of markers in samples. If it is a weak expression, It might be hard to be detected in clinical diagnosis. Therefore, we searched these filtered lncRNAs included in this study in TIMER: Tumor IMmune Estimation Resource (http://timer.cistrome.org/) (Zhu Y. et al., 2021).

#### 2.2.4. Immune Landscapes Assessment of the Prognostic Signature

In order to explore the immune landscapes of the high-risk group and low-risk group, we analyzed the correlation between risk score and immune cells by Spearman analysis. The R Package “Limma” was used to filter the immune cell infiltration of samples with different risk scores. In addition, we analyzed the expression levels of 29 immune checkpoint moleclues in samples from different risk groups, including CD70, TNFSF9, FGL1, CD276, NT5E, HHLA2, VTCN1, TNFRSF18, CD274, PDCDL1LG2, IDO1, CTLA4, ICOS, HAVCR, PDCD1, LAG3, SIGLEC15, TNFSF4, TNFRSF9, ENTPD1, VSIR, ICOSLG, TNFSF14, TMIGD2, CD27, NCR3, BTLA, CD40LG, CD40, TNFRSF4.

#### 2.2.5. Simple Nucleotide Variations of the Prognostic Signature

Based on the tumor mutational burden (TMB) information of each sample (Bonfield et al., 2021), combined with the survival information analysis of patients, all samples were divided into the high-TMB group and the low-TMB group, and the survival of the two groups was analyzed. The survival of the high-TMB + high-risk group, the high-TMB + low-risk group, the low-TMB + high-risk group and the low-TMB + low-risk score group were jointly analyzed to observe the influence of the risk score and TMB on the survival of the patients. Genes with significant mutation rates in the high-risk group and low-risk group were further analyzed to find the ones with the most significant mutation rates for further analysis.

#### 2.2.6. Prognostic Analysis and Immune Infiltration of Single lncRNA

Based on the expression levels of each independent lncRNA in the 6 m^6^A/m^5^C/m^1^A-related lncRNAs Prognostic signatures, the patient samples were divided into high expression group and low expression group. The R package “survival” was used to obtain survival curves for each independent lncRNA. There are six types of immune infiltration in human cancers, including Wound Healing (Immune C1), IFN-gamma Dominant (Immune C2), Inflammatory (Immune C3), Lymphocyte Depleted (Immune C4), Immunologically Quiet (Immune C5) and TGF-beta Dominant (Immune C6) (Soldevilla et al., 2019; Zhao et al., 2021). Based on the relevant files downloaded from the UCSC Xena database, the R package “CIBERSORT” and “ESTIMATE” (Wang S. et al., 2020) were used to obtain relevant data (Stromal Score, Immune Score, ESTIMAT Score) of tumor purity. Furthermore, the correlation between the expression levels of 6 m^6^A/m^5^C/m^1^A-related lncRNAs and tumor purity in the immune microenvironment of HNSCC was further analyzed.

#### 2.2.7. Drug Sensitivity Analysis

The R package “Prophetic” was used to analyze the drug sensitivity analysis of samples from different risk groups (Ding et al., 2021). Combined with the relevant data downloaded from the CellMiner Database, the correlation analysis of drug sensitivity and the expression level of 6 m^6^A/m^5^C/m^1^A-related lncRNAs was further carried out (Xu X. et al., 2020).

## 3. Results

### 3.1. m^6^A/m^5^C/m^1^A-Related lncRNAs

**FIGURE 1:**
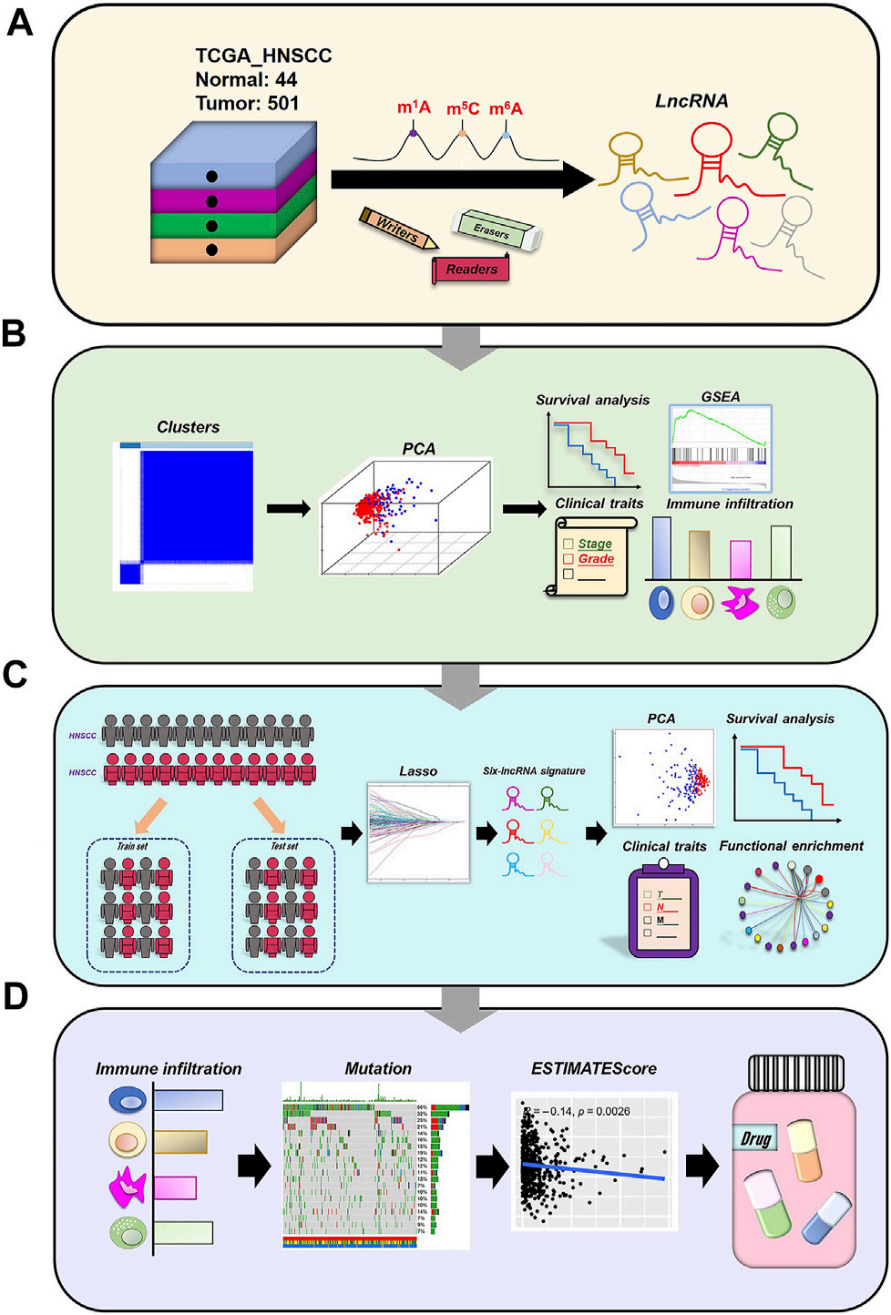
Analysis flow chart.

The flow chart of our work was shown in Figure 1.

**FIGURE 2:**
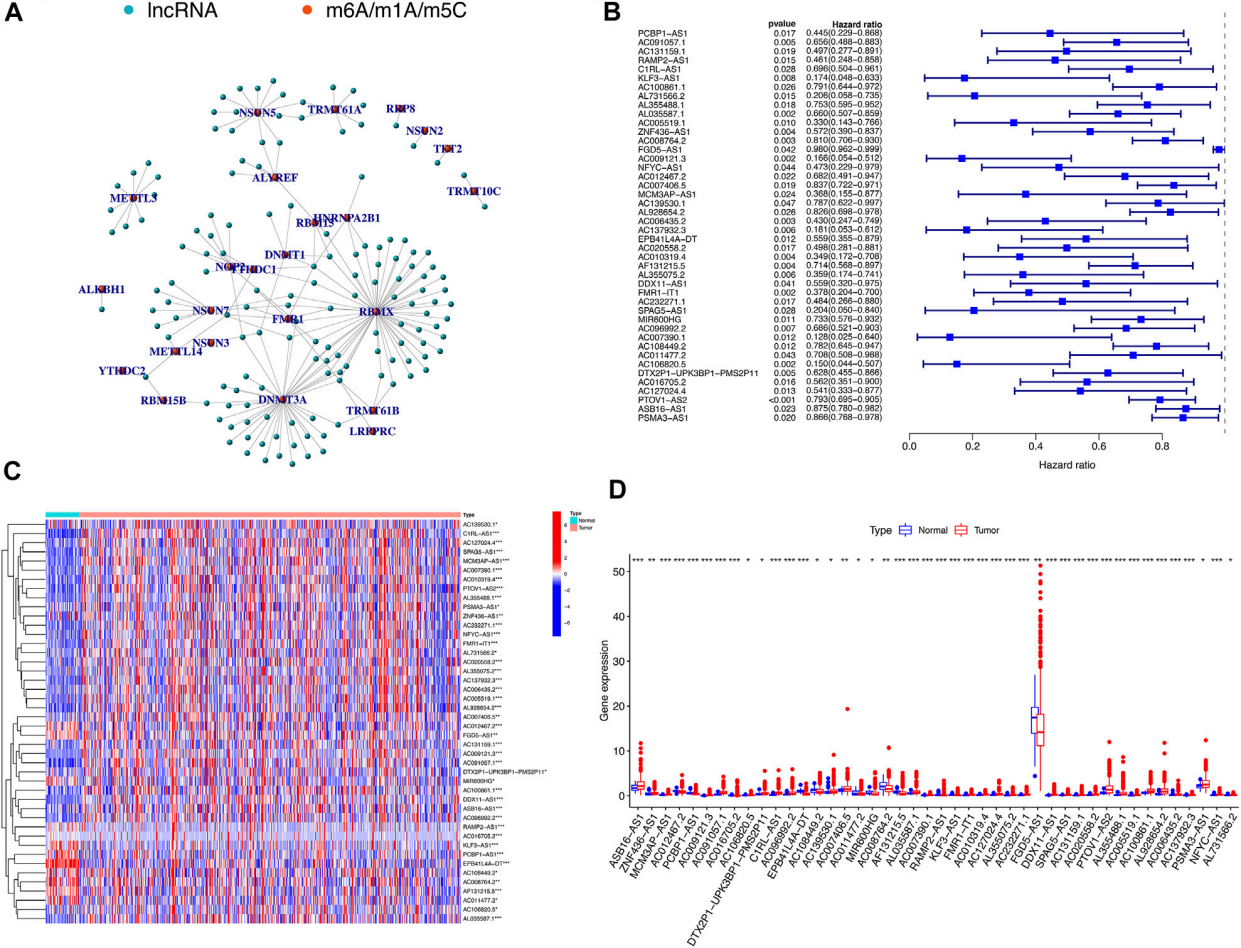
m^6^A/m^5^C/m^1^A-related mRNA–lncRNA co-expression network and identification of prognostic value of m^6^A/m^5^C/m^1^A-related lncRNAs. **(A)** 147 lncRNAs co-expressed with m^6^A/m^5^C/m^1^A-related genes. Green represents lncRNAs, while red represents m^6^A/m^5^C/m^1^A-related genes. **(B)** The forest plot of univariate Cox regression showed that 44 lncRNAs with prognostic value were protective factors of HNSCC. **(C,D)** The heatmap and boxplot of differential expression of 44 lncRNAs with prognostic value between 44 normal tissues and 501 tumor tissues in TCGA-HNSCC database.

Using the downloaded file from the “GENCODE” website, we obtained 14,086 lncRNAs expression matrix. In addition, 52 expression matrix of m^6^A/m^5^C/m^1^A-related genes were screened out from the expression matrix of mRNAs. Through co-expression analysis, we screened out 147 lncRNAs co-expressed with m^6^A/m^5^C/m^1^A-related genes (Figure 2A). We further screened out 44 lncRNAs with potential prognostic value from 147 co-expressed lncRNAs. The forest plot showed that all 44 lncRNAs were protective factors (hazard ratio < 1) in patients with HNSCC (Figure 2B). The heatmap and box figures show that the expressions of 44 lncRNAs in normal samples and tumor samples are significantly different (Figures 2C,D).

### 3.2. Consensus Clustering of m^6^A/m^5^C/m^1^A-Related lncRNAs Identified Two Clusters of HNSCC Patients

**FIGURE 3:**
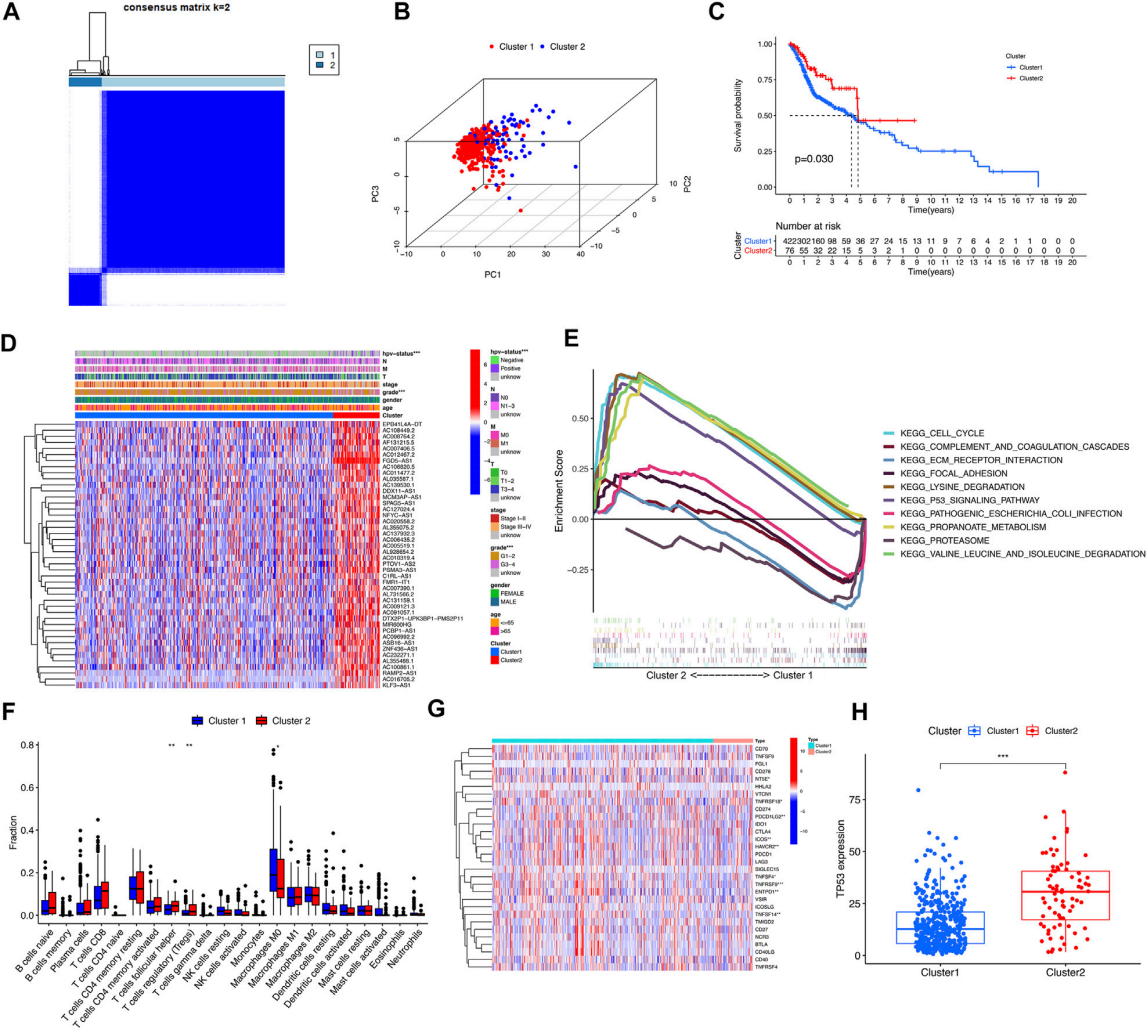
Clinical characteristics and overall survival among different subgroups of HNSCC. *, **, *** represent *p* < 0.05, *p* < 0.01 and *p* < 0.0001, respectively. **(A)** Consensus matrix for optimal k = 2. **(B)** Principal component analysis (PCA) of cluster 1 and cluster 2. **(C)** Kaplan-Meier curve of overall survival (OS) time in Cluster 1 and Cluster 2 (*p* = 0.03). **(D)** Heatmap of the clinical features of the two clusters. **(E)** GSEA analysis displayed key pathways in in Cluster 1 and Cluster 2. **(F)** The violet plot showed the differential expression of immune cells between Cluster 1 and Cluster 2, in which Cluster 1 was represented in blue and Cluster 2 was represented in red. **(G)** The heatmap of the differential expression levels of 29 immune checkpoint genes between Cluster 1 and Cluster 2. **(H)** The boxplot of TP53 expression level between Cluster 1 and Cluster 2.

By cluster analysis, the tumor samples in the TCGA database were classified into two types, consensus matrix for optimal K = 2 (Figure 3A). PCA was used to represent the characteristics of the typing samples, and it was found that the samples could be visually distinguished (Figure 3B). The Kaplan-Meier survival curves showed that the prognosis of patients in Cluster 2 was significantly better than that of patients in Cluster 1 (*p* = 0.03) (Figure 3C), and the two clinical characteristics of patients’ HPV status and tumor grade were significantly different among different types (*p* < 0.001) (Figure 3D). GSEA analysis showed that the pathways of Cluster 2 were mainly enriched in CELL_CYCLE, LYSINE_DEGRADATION, P53_SIGNALING_PATHWAY, et al. The functional enrichment pathways of Cluster1 were mainly enriched in ECM_receptor_interaction, FOCAL_ADHESION, OCAL_ADHESION, et al. (Figure 3E). According to the immune microenvironments of the two subtypes, we can found the infiltration of T cells follicular helper and T cells regulatory in Cluster 1 significantly less than Cluster 2 (*p* = 0.005; *p* = 0.009), while Macrophages M0 infiltrate significantly more than Cluster 2 (*p* = 0.05) (Figure 3F). We found that the expression levels of 9 immune checkpoint genes were significantly different between the two subtypes (*p* < 0.05). The expression levels of NT5E, ICOS, PDCD1LG2, HAVCR2, TNFSF4, TNFRSF9, EntPD1 and TNFSF14 were higher in Cluster 1, and TNFRSF18 was higher in Cluster 2 (Figure 3G). In addition, the expression level of tumor suppressor gene TP53 in Cluster 2 was significantly higher than that in Cluster 1 (*p* < 0.001) (Figure 3H).

### 3.3. The Risk Prognostic Signature Built by m^6^A/m^5^C/m^1^A-Related lncRNAs

**FIGURE 4:**
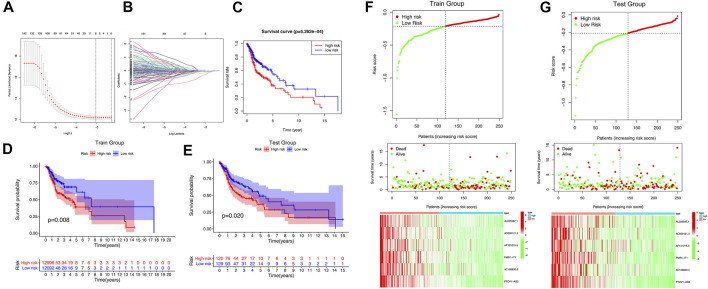
The m^6^A/m^5^C/m^1^A-related lncRNAs Prognostic signature. **(A,B)** 6 m^6^A/m^5^C/m^1^A-related lncRNAs were found by using least absolute shrinkage and selection operator (LASSO) cox regression. **(C)** Kaplan-Meier curve of overall survival of high-risk group and low-risk group. **(D,E)** Kaplan-Meier curve of overall survival of train group and test group. **(F,G)** The distribution of risk score, survival status and m^6^A/m^5^C/m^1^A-related lncRNAs expression panal.

We got 44 lncRNAs with prognostic value, but it is too difficult to use all lncRNAs to build a prognostic model, so we compressed 44 lncRNAs with Lasso analysis method, and finally got 6 lncRNAs with the most prognostic value to build our prognostic model: AL035587.1, AC009121.3, AF131215.5, FMR1-IT1, AC106820.5, PTOV1-AS2 (Figures 4A,B). The correlation coefficient was shown in Table 2.

**TABLE 2:** The correlation coefficient of m^6^A/m^5^C/m^1^A-related lncRNAs.

Gene	Coef
AL035587.1	−0.0556622865128334
AC009121.3	−0.0032158199257021
AF131215.5	−0.015569033248601
FMR1-IT1	−0.130840197918301
AC106820.5	−0.320536009497966
PTOV1-AS2	−0.0694514535421893

The survival time and survival rate of patients in the high-risk subgroup were significantly lower than those in the low-risk subgroup (*p* = 5.262e-04) (Figure 4C), which was also verified in samples from the training set and the testing set (Train group: *p* = 0.008; Test group: *p* = 0.02) (Figures 4D,E). It was found in the training set and testing set that the prognosis of patients in the high-risk subgroup was worse than that in the low-risk subgroup, and the expression levels of 6 lncRNAs in the low-risk subgroup were significantly higher than that in the high-risk subgroup (Figures 4F,G). The univariate and multivariate regression analysis results showed that risk score, as an independent prognostic indicator, had a certain predictive value relative to individual and all clinical characteristics (age, gender, tumor grade, tumor stage) in both the training set and the testing set (*p* < 0.01) (Supplementary Figures S1A–D). The results of PCA and T-SNE show that the use of risk score can intuitively distinguish the high-risk group from the low-risk group in the training set and the testing set (Supplementary Figures S1E–H). As shown in Supplementary Figure S2, the area under curve (AUC) at 5 years of overall survival for the LncSig (our study) is 0.607, which was significantly higher than that of JianglncSig (AUC = 0.602) and WanglncSig (AUC = 0.589). These results indicate that LncSig has better prognostic performance in predicting survival than the two recently published lncRNA signatures (Wang et al., 2018; Jiang et al., 2020).

**FIGURE 5:**
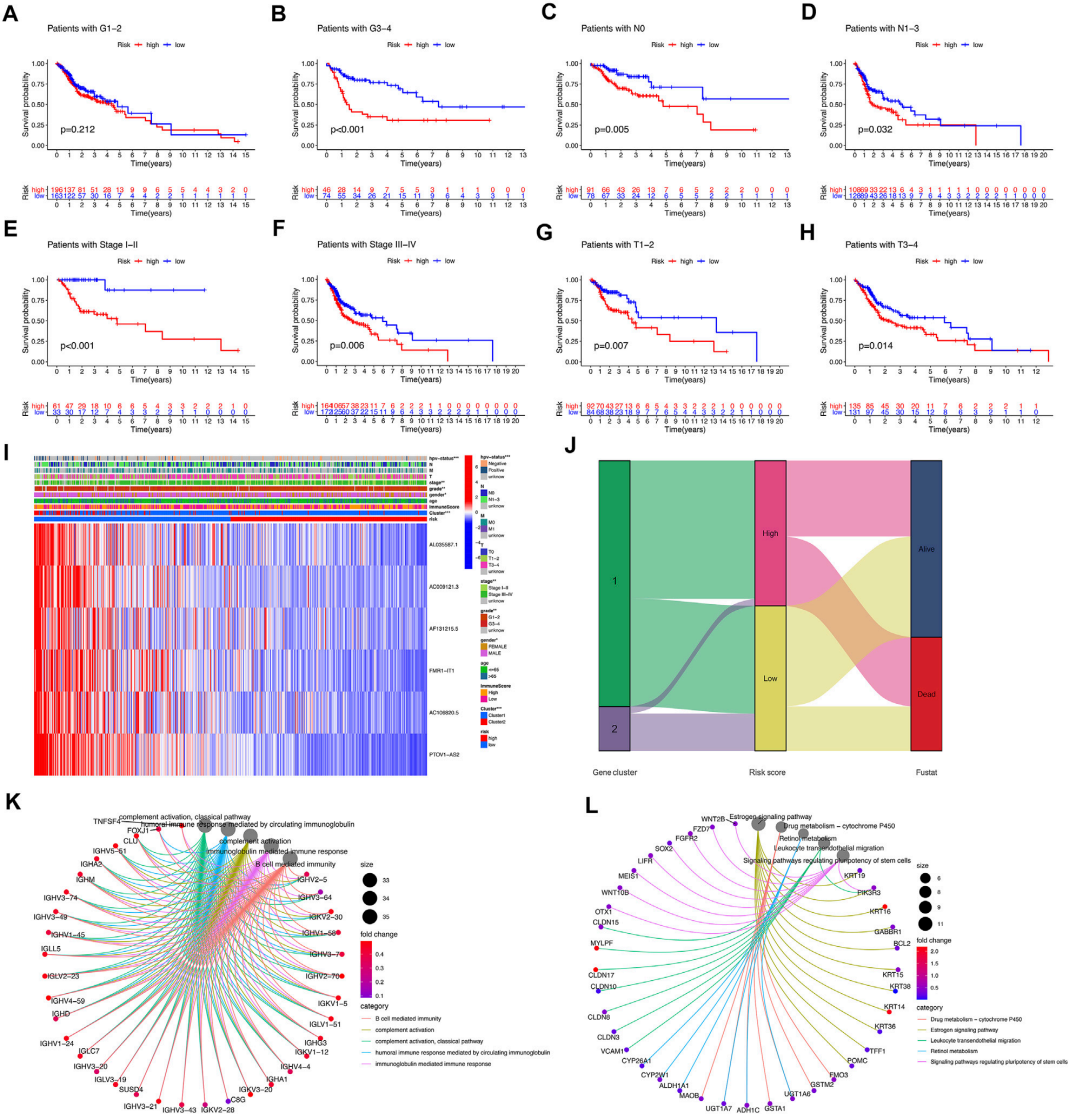
Correlation of risk model subgroups with overall survival and different clinical characteristics in TCGA-HNSCC database. **(A–H)** Kaplan–Meier curve of the overall survival of patients with different clinical traits in high-risk group and low-risk group. **(I)** Heatmap of association between expression levels of six prognosis lncRNAs and clinicopathological features in TCGA database. **(J)** The sankey diagram of the relationship among the cluster, risk score and survival state. **(K,L)** GO and KEGG analysis.

In combination with clinical features, analysis showed that patients in the tumor grade G3-4, N0, N1-3, stage I–II, stage III–IV, T1-2, T3-4, the survival of high-risk subgroup was significantly worse than low-risk subgroup (*p* < 0.05) (Figures 5A–H). The heatmap showed the expression of 6 selected lncRNAs in patients with high and low risk. We observed the statistically significant differences between the high-risk and low-risk groups in gender, HPV-status, tumor stage and tumor grade (*p* < 0.05) (Figure 5I). The results of sankey diagram showed that most of the patients in Cluster 2 belonged to the low-risk group and had a higher survival rate than Cluster 1, which also supported the results of survival status analysis of the above classifications (Figure 5J). GO analysis showed that cell functions enriched by different genes between the high-risk group and the low-risk group were as follows: B cell mediated immunity, complement activation, complement activation classical pathway, humoral immune response mediated by circulating immunoglobulin, immunoglobulin mediated immune response (Figure 5K). KEGG analysis showed that the signal pathways enriched by differential genes between the high-risk group and the low-risk group were: drug metabolism-cytochrome P450, estrogen signaling pathway, leukocyte transendothelial migration, retinol metabolism, signaling pathway regulating pluripotency of stem cells (Figure 5L).

The gene expression box plots (Supplementary Figure S3) of FMR1-IT1 and PTOV1-AS2 were found in the Module of exploring associations between gene expression and tumor features in TCGA from TIMER, which showed that the expression of FMR1-IT1 and PTOV1-AS2 in HSNCC tissues is significantly higher than that in normal tissues.

### 3.4. Immune Landscapes and Tumor Mutational Burden (TMB) of the Prognostic Signature

**FIGURE 6:**
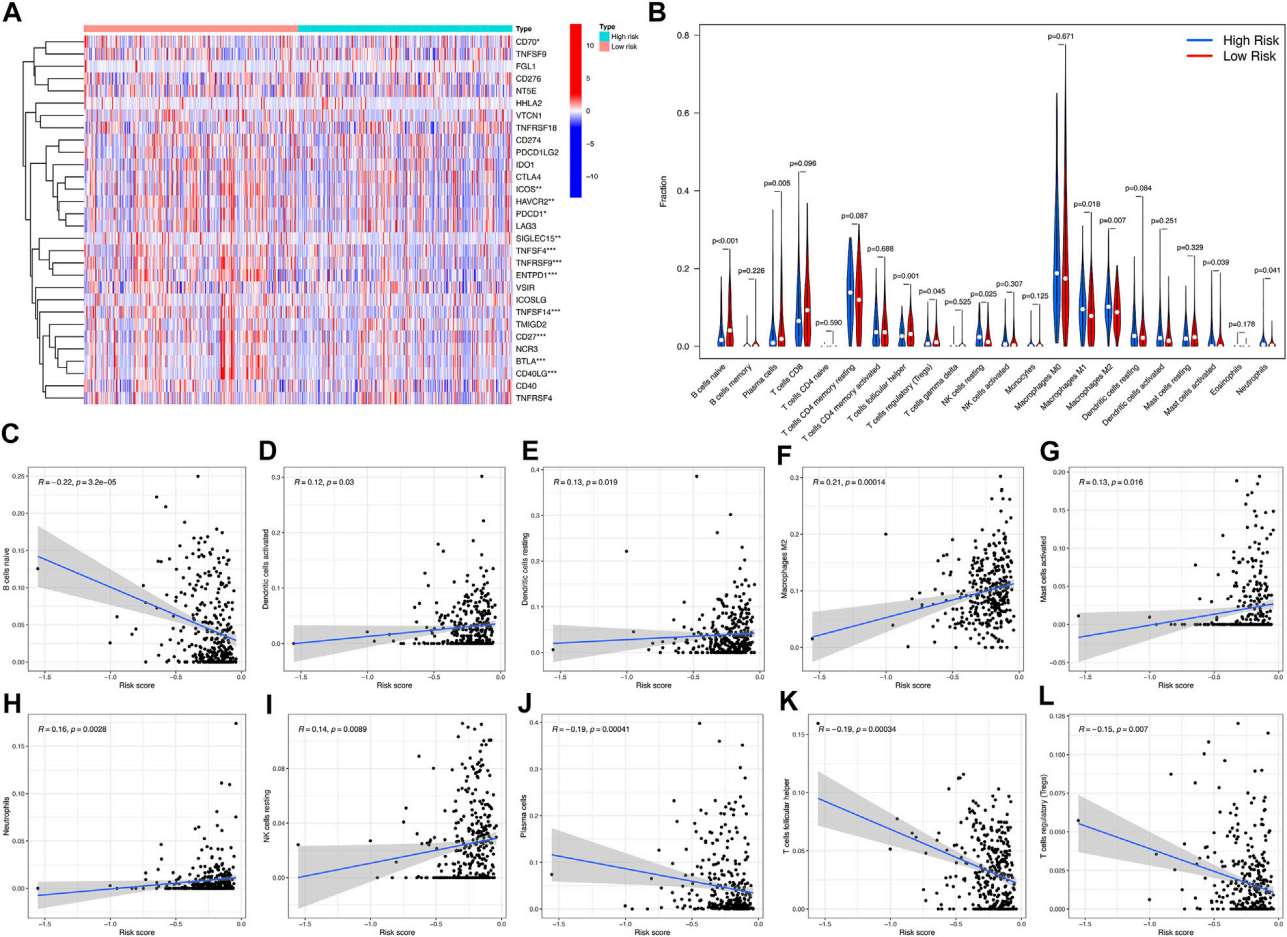
Differences in immune microenvironment between high-risk and low-risk subgroups. **(A)** Violet plot of the expression of different checkpoint genes between high-risk subgroup and low-risk subgroup. **(B)** Heatmap of infiltration of different immune cells between high-risk subgroup and low-risk subgroup. **(C–L)** The correlation between immune cell infiltration and risk score.

We found that 12 immune checkpoint genes were significantly different between the two risk groups (*p* < 0.05). CD70, ICOS, HAVCR2, PDCD1, SIGLEC15, TNFSF4, TNFRSF9, ENTPD1, TNFSF14, CD27, BTLA, CD40LG. The expression of BTLA was higher in low-risk group. CD70, ICOS, HAVCR2, PDCD1, SIGLEC15, TNFSF4, TNFRSF9, ENTPD1, TNFSF14, CD27 and CD40LG were higher expressed in high-risk group (Figure 6A). Among them, the overlapping immune checkpoint genes ICOS, HAVCR2, TNFSF4, TNFRSF9, ENTPD1 and TNFSF14 were all increased in Cluster 1 and high-risk subgroups.

Immunity microenvironment analysis showed that the contents of NK cell resting, macrophages M2, and neutrophils in samples of low-risk group were significantly lower than those of high-risk group (*p* < 0.05), while the contents of B cells navie, plasma cells, and T cells regulatory (Tregs) were on the contrary (*p* < 0.05) (Figure 6B). The correlation analysis between risk score and immune cell infiltration showed that neutrophils, dendritic cells activated, NK cells resting, dendritic cells resting, macrophages M2, mast cells activated were positively correlated with risk score. B cells naive, plasma cells, T cells helper and T cells regulatory (Tregs) were negatively correlated with risk score (*p* < 0.05) (Figures 6C–L).

**FIGURE 7:**
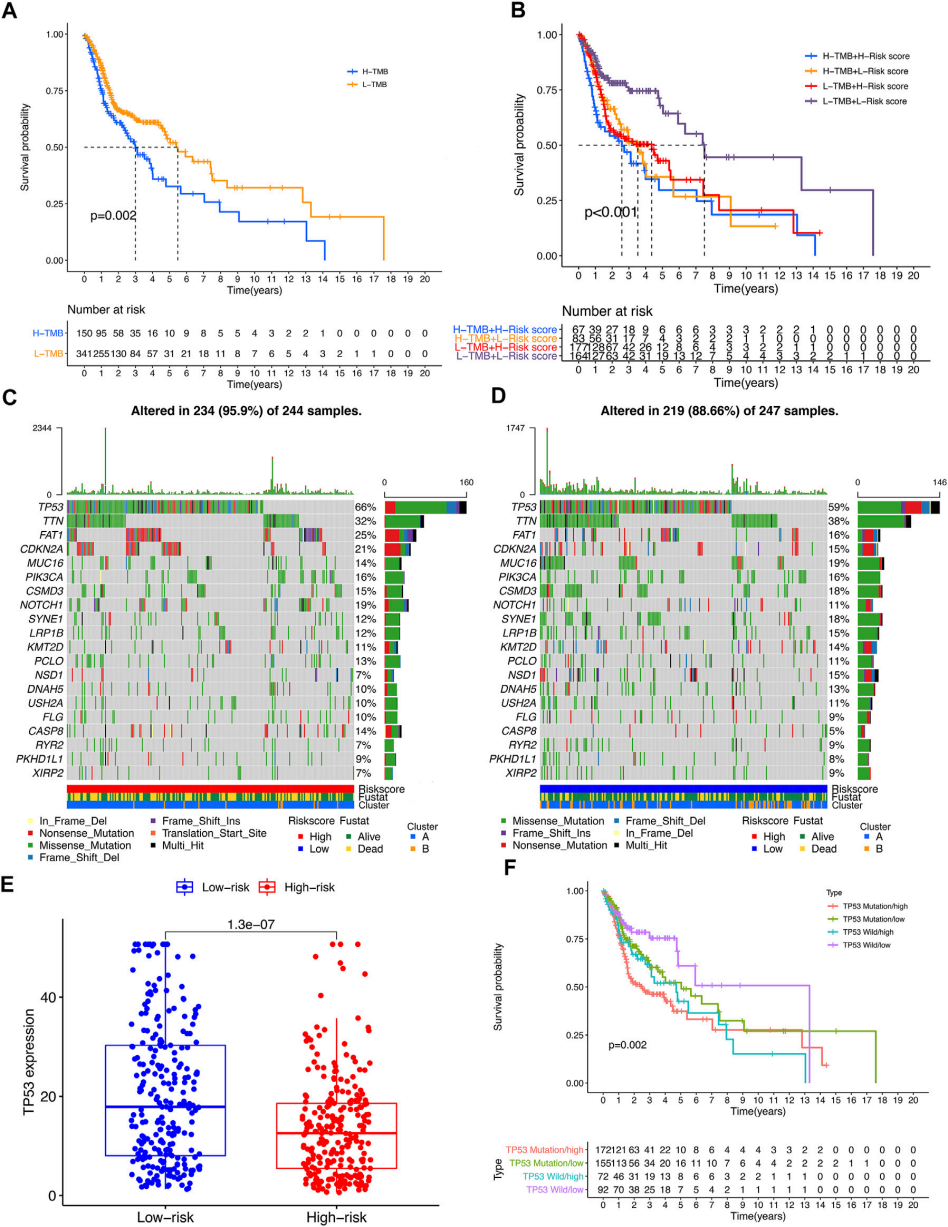
The impact of differential TMB level on patients’ prognosis and TP53 somatic mutation. **(A)** Comparison of prognosis between high TMB subgroup and low TMB subgroup. **(B)** Comparison of prognosis of TMB combined with risk score. **(C,D)** Top 20 genes with the most significant mutation rates in high-risk subgroup and low-risk subgroup. **(E)** The differential expression level TP53 between high-risk group and low-risk group. **(F)** Kaplan–Meier curve analysis of overall survival was shown for patients classified according to TP53 mutation status and risk score. TP53-wt: wild type of TP53 sequence; TP53-mt: mutation type of TP53 sequence.

TMB analysis showed that the survival of the high-TMB group was significantly lower than that of the low-TMB group (*p* = 0.002) (Figure 7A). Combined analysis of risk score showed that patients in the low-TMB + low-risk score group had a significantly better prognosis than those in the other three groups (*p* < 0.001), suggesting that patients with low TMB tend to have a better prognosis (Figure 7B). Looking for the significant mutant genes in the high-risk group and the low-risk group, it was found that the mutation rate of TP53 was the highest in both groups, and the expression of TP53 in the high-risk group was significantly lower than that in the low-risk group (*p* = 1.3e-07) (Figures 7C–E). The combined analysis of TP53 and risk score showed that the expression level of TP53 in the high-risk group was higher than that in the low-risk group (*p* = 1.3e-07). Patients in the low-risk subgroup of TP53 wild-type had significantly better outcomes than those in the other three groups (*p* = 0.002) (Figure 7F).

### 3.5. Prognostic Analysis and Immune Infiltration of m^6^A/m^5^C/m^1^A-Related lncRNAs

**FIGURE 8:**
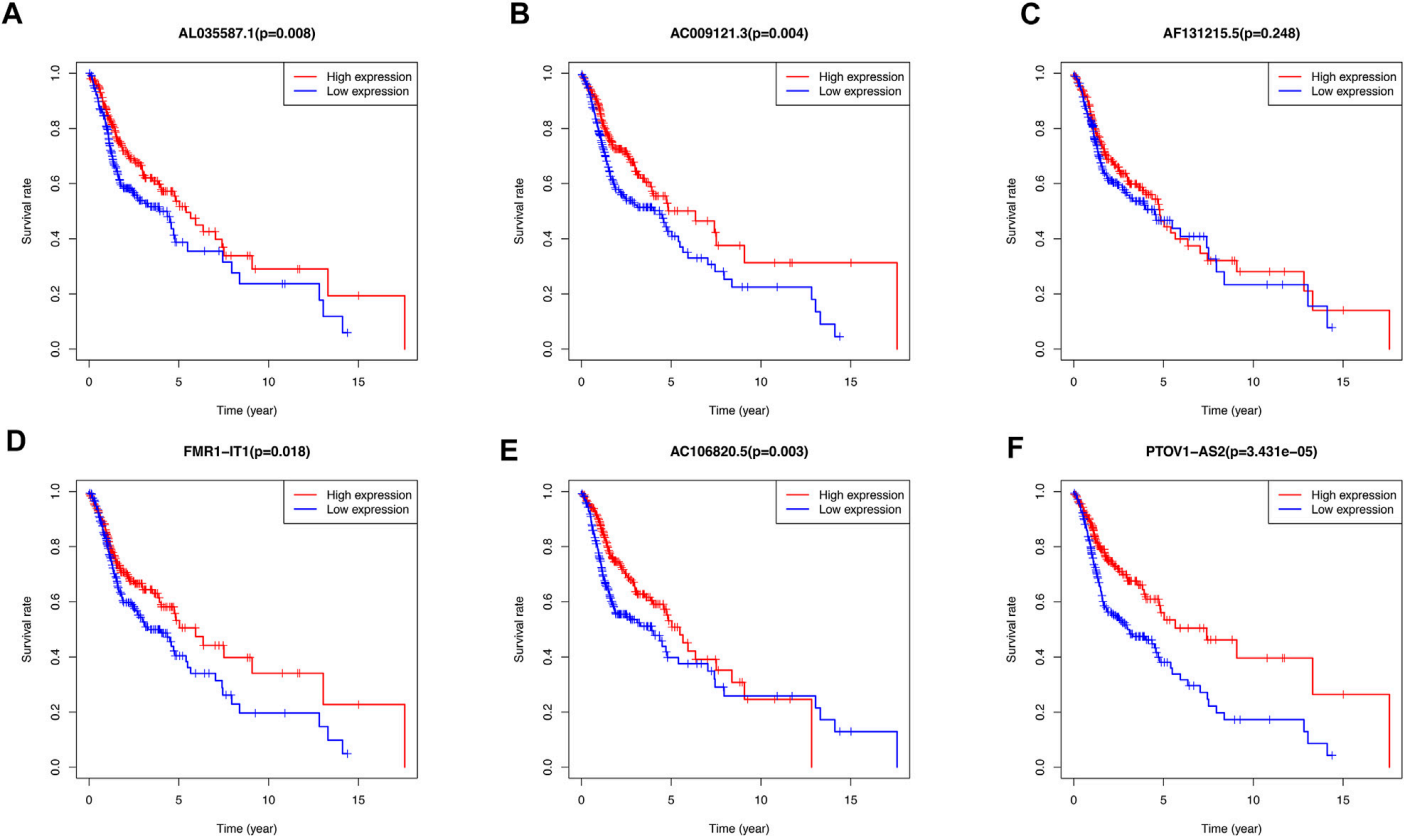
Kaplan-Meier curves of overall survival for differential expression level of 6 m^6^A/m^5^C/m^1^A-related lncRNAs Prognostic signatures respectively. **(A)** The association of AL035587.1 expression level with patients’ prognosis of TCGA database. **(B)** The association of AC009121.3 expression level with patients’ prognosis of TCGA database. **(C)** The association of AF131215.5 expression level with patients’ prognosis of TCGA database. **(D)** The association of FMR1-IT1 expression level with patients’ prognosis of TCGA database. **(E)** The association of AC106820.5 expression level with patients’ prognosis of TCGA database. **(F)** The association of PTOV1-AS2 expression level with patients’ prognosis of TCGA database.

The Kaplan-Meier survival curves showed that there was no significant difference between high-expression and low-expression of AF131215.5. However, the high-expression of AL035587.1, AC009121.3, FMR1-IT1, AC106820.5, PTOV1-AS2 had the better overall survival than low-expression in TCGA database (*p* < 0.05) (Figures 8A–F).

**FIGURE 9:**
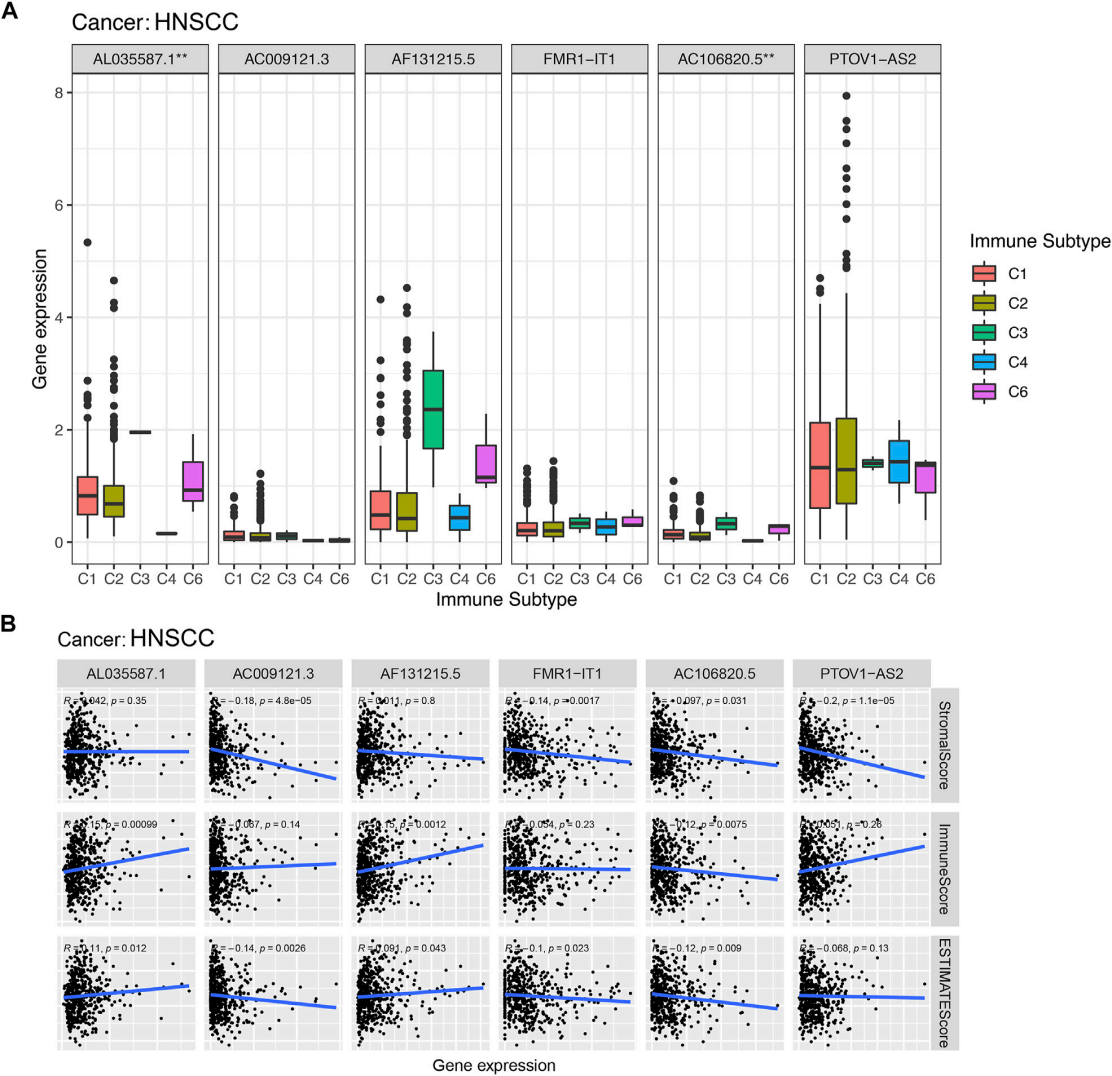
Association of expression of prognostic lncRNAs with with immune response, TME. *, **, *** represent *p* < 0.05, *p* < 0.01 and *p* < 0.0001, respectively. **(A)** Association of prognostic lncRNAs expression with immune subtypes in HNSCC patients. **(B)** Correlation of expression of prognostic lncRNAs with TME (Stromal score, Immune score, and ESTIMATE score).

According to the results of the immunoinfiltration analysis, the high expression of AL035587.1 was considered to be related to the type of C3 and C6 infiltration according to the results of Figure 9A. The high expression of AC106820.5 was considered to be correlated with the type of C3 and C6 infiltration (*p* < 0.01). Moreover, the tumor microenvironment (TME) was assessed by estimation analysis. The expression levels of PTOV1-AS2, AC009121.3, FMR1-IT1 and AC106820.5 were negatively correlated with the stromalscore (Figure 9B). In addition, AL035587.1 and AF131215.5 were positively correlated with immunescore, while AC106820.5 was negatively correlated. Estimatescore (combining stromalscore and immunescore) showed that the expression levels of AC009121.3, AC106820.5 and FMR1-IT1 were negatively correlated with estimatescore, while AL035587.1 and AF131215.5 were positively correlated (Figure 9B) (*p* < 0.05).

### 3.6. Drug Sensitivity

**FIGURE 10:**
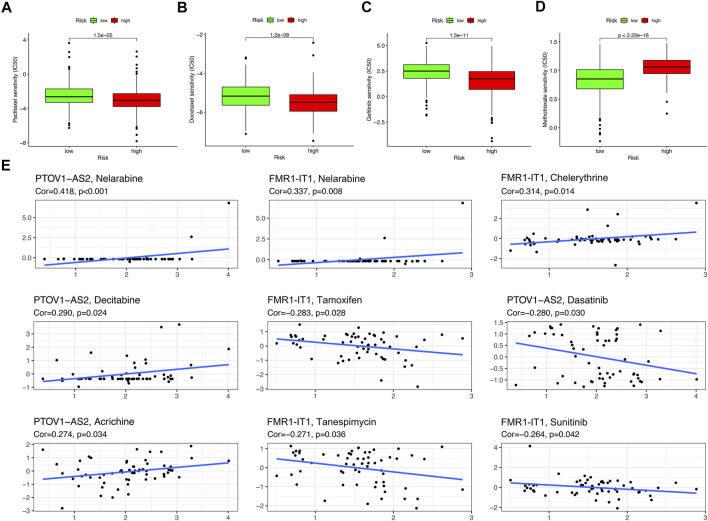
Drug sensitivity analysis. **(A–D)** The boxplot of Sensitivity of Paclitaxel, Docetaxel, Gefitinib, Methotrexate in patients with high-risk subgroup and low-risk subgroup. **(E)** The scatter plot showed the correlation between prognostic lncRNAs expression and drug sensitivity (Z score of CellMiner interface). Top 10 associations were displayed, sorted by *p* value.

Drug sensitivity analysis showed that samples from the low-risk group had higher sensitivity to Paclitaxel, Docetaxel and Gefitinib and lower sensitivity to Methotrexate than those from the high-risk group (Figures 10A–D) (*p* < 0.001). Two m^6^A/m^5^C/m^1^A-related lncRNAs: PTOV1-AS2 and FMR1-IT1 were extracted from the CellMiner Database NCI 60 RNA seq and Compound activity: DTP NCI-60. It was found that the sensitivity of drug Nelarabine had the highest correlation with the expression level of PTOV1-AS2 (Correlation = 0.418, *p* < 0.001), and the sensitivity of Nelarabine was also the highest associated with FMR1-IT1 expression (Correlation = 0.337, *p* = 0.008) (Figure 10E).

## 4. Discussion

Studies have shown that dynamic RNA methylation, the modification events (such as m^6^A, m^5^C, and m^1^A) are involved in the progression of tumors, including promoting cancer cell proliferation or regulating invasiveness and metastatic potential (Barbieri and Kouzarides, 2020). m^6^A can also be involved in tumor proliferation, migration, drug resistance, or as a prognostic marker of tumor (Han et al., 2019; Wang M. et al., 2020; Wang H. et al., 2021; Paramasivam and Priyadharsini, 2021; Xu et al., 2021). Some studies have shown that there is an interaction between RNA methylation and lncRNA in tumors, and m^6^A reader YTHDF2-mediated degradation of lncRNA FENDRR promoted cell proliferation in endometrioid endometrial carcinoma (Shen et al., 2021). m^6^A-mediated upregulation of LINC00857 promoted pancreatic cancer tumorigenesis (Meng L. et al., 2021). LncRNA KB-1980E6.3 maintained breast cancer stem cell stemness via interacting with IGF2BP1 (Zhu P. et al., 2021). Abnormal m^5^C modification of lncRNA H19 mediated by NSUN2 was associated with poor differentiation of hepatocellular carcinoma (Sun et al., 2020). Conversely, lncRNAs can also affect the function of genes related to RNA methylation modifications. LncRNA MALAT1 contributed to thyroid cancer progression through the upregulation of IGF2BP2 by binding to miR-204 (Ye et al., 2021). LncRNA LINRIS can stabilize IGF2BP2 and promote aerobic glycolysis in colorectal cancer (Wang et al., 2019). The above evidences indicate the interaction between RNA methylation modifications and lncRNAs in the tumorigenesis of a variety of cancers. However, the evidence of how the RNA methylation modification involved in the development of HNSCC by influencing the lncRNA is still insufficient. Therefore, this study aims to explore whether the relevant lncRNAs are involved in the development of the HNSCC.

We downloaded the data of 44 Normal samples and 501 tumor samples from the TCGA database to search for m^6^A/m^5^C/m^1^A-related lncRNAs and we found 44 lncRNAs with prognostic value. Tumor samples were divided into Cluster 1 and Cluster 2 according to the expression levels of these 44 lncRNAs in all tumor samples, and the survival of Cluster 2 was significantly better than that of Cluster 1. We further used the lasso cox regression to construct the prognostic signature using 6 m^6^A/m^5^C/m^1^A-related lncRNAs to predict survival in patients with HNSCC. Based on the correlation coefficients of the 6 m^6^A/m^5^C/m^1^A-related lncRNAs and their expression levels in each tumor sample, we obtained risk scores for each tumor sample and constructed a prognostic signature. According to Figure 5J the sankey diagram of the relationship among the cluster, risk score and survival state, it can be found that most patients in high risk group come from cluster 1, while most patients in cluster 2 are assigned to low risk group. The survival time of patients in the high risk group was significantly shorter than that in the low risk group (*p* < 0.05), which also proved that both the clustering type composed of 44 lncRNAs and the prognosis model composed of 6 lncRNAs can well predict the prognosis of tumor patients. Differences in survival were also demonstrated in the training and validation sets of randomly assigned tumor samples. The univariate and multivariate cox regression analysis showed that risk score could be used as a prognostic marker for HNSCC independent of age, sex, tumor grade, and tumor stage. This suggested that the prognostic signature constructed by 6 lncRNAs had certain prognostic value. These results supply a reference for further functions and mechanisms of prognostic signatures in HNSCC.

Six m^6^A/m^5^C/m^1^A-related lncRNAs were obtained, which were AL035587.1, AC009121.3, AF131215.5, FMR1-IT1, AC106820.5 and PTOV1-AS2. The results in Supplementary Figure S3 showed that FMR1-IT1 and PTOV1-AS2 can be detected in clinical diagnosis. AF131215.5 is one of lncRNAs in lung adenocarcinoma (LUAD)-related competing endogenous RNA networks, and showed an independent prognostic value of overall survival for patients with LUAD (Hou and Yao, 2021). FMR1 intronic transcript 1 (FMR1-IT1) is also known as FMR1 intronic transcript 1 (non-protein coding) or Fragile X Mental Retardation Syndrome Protein (FMRP) or FMR1, there were evidence that FMRP regulated tumor invasiveness-related pathways in melanoma cells by impacting cell migration, invasion and adhesion (Zalfa et al., 2017), and its overexpression was associated with lung metastasis of murine breast cancer (Lucá et al., 2013). However, there is still not enough evidence to clarify the relationship between tumorigenesis and prognosis of these 6 lncRNAs and HNSCC. We hope that our work can explain the role of these 6 lncRNAs in the carcinogenesis and development of HNSCC and clarify their prognostic value.

Based on the expression levels of these 6 m^6^A/m^5^C/m^1^A-related lncRNAs in the samples, we further studied the tumor microenvironment of the samples and analyzed the expression levels of 29 immune checkpoint genes. There were significant differences in the expression of various immune cells and immune checkpoint genes between the high-risk subgroup and the low-risk subgroup. Among them, the overlapping immune checkpoint genes of Cluster 1 and high-risk subgroups were ICOS, HAVCR2, TNFSF4, TNFRSF9, ENTPD1 and TNFSF14. Their expression levels were all elevated, revealing the characteristics of tumor immune microenvironment between different subgroups. Currently, only a small number of HNSCC patients benefit from immunotherapy, and the need to discover new biomarkers to optimize treatment strategies has become increasingly important in clinical practice. With the rise of the application of immune checkpoint inhibitor in tumor treatment, such as Programmed Cell death-1 (PD-1) inhibitors (Kitamura et al., 2020), there are many ongoing trials in HNSCC that focus on identifying new biomarkers (Gavrielatou et al., 2020). As a member of the B7 superfamily, ICOS is a co-stimulatory receptor for T-cell enhancement. In preclinical studies, ICOS agonist monoclonal antibodies have been shown to enhance inhibitory checkpoint blockade. On the contrary, the antagonistic monoclonal antibody against ICOS can not only inhibit the lymphoid tumor cells expressing ICOS, but also inhibit the immunosuppressive Treg (Amatore et al., 2018). ENTPD1 (CD39) converts extracellular ATP into adenosine, thereby inhibiting T cell effector functions through the adenosine receptor A2A. The CD39 inhibitor POM-1 inhibits adenosine production and reduces T cell suppression. The adenosine pathway can be targeted to multiple myeloma, and blocking this pathway can replace PD1/PDL1 to inhibit multiple myeloma and other blood system cancers (Yang et al., 2020). At present, TNFSF14-based therapy has shown great efficacy in cancer immunotherapy, which can modify tumor microenvironment by normalizing tumor vessels and significantly improve the infiltration of effector infiltrating lymphocytes, thus reducing tumor burden and forming lasting anti-tumor memory (Skeate et al., 2020). These evidences also suggest the possibility of selecting differential immune checkpoint genes as therapeutic targets for HNSCC. In addition, we have analyzed the joint tumor mutation burden and risk score model. It was found that the prognosis of patients with high burden of tumor mutations was worse than that of patients with low burden, while the prognosis of patients with high burden of mutations in the high-risk subgroup was the worst, and the prognosis of patients with low burden of mutations in the low-risk subgroup was the best. This undoubtedly indicated the importance of tumor mutation burden in predicting prognosis. Moreover, we observed that the tumor suppressor gene TP53 was highly expressed in Cluster 2 and the low-risk subgroup. It is generally believed that TP53 gene can be divided into wild type and mutant type. The wild-type TP53 gene, namely tumor suppressor gene, has a control and negative regulation effect on cell proliferation. However, the mutant TP53 gene lost its tumor suppressor effect and promoted the transformation of cells to malignancy (Duffy et al., 2020; Zhu et al., 2020; Meng X. et al., 2021). Our results showed that TP53 mutant patients in the high-risk subgroup had the worst prognosis, and TP53 wild-type patients in the low-risk subgroup had the best prognosis, which also indicated that higher mutation rate of TP53 was associated with poorer prognosis. In addition, some studies have revealed the connection between lncRNA and TP53. Huarte, M, et al. found that LincRNA-p21 is the first lncRNAs identified as being induced by wt-p53 transcription. It acts as a transcriptional repressor in the p53 pathway (Huarte et al., 2010). Chang et al. (2011) found that lncRNA XIST and the p53-inducible lncRNA NEAT1 act as ceRNA for miR-34a, which leads to the activation of epithelial-mesenchymal transition. The research of Olivero et al. (2020) showed that TP53 activates the lncRNA pvt1b to inhibit myelocytomasis and suppress tumorigenesis. It is worth noting that targeting mut-TP53/ncRNA signals may have a great potential impact on the treatment of cancer patients. Interestingly, lncRNA and immune checkpoint are also closely related, lncRNAs are involved in the regulation of various cellular pathways and immune checkpoints (Di Martino et al., 2021). Tang et al. (2017) found that lncRNA AFAP1-AS1 was co-expressed with PD-1 lymphocytes associated with nasopharyngeal carcinoma, which resulted in invasive and poor prognosis phenotype. In hepatocellular carcinoma, lncRNA Tim3 mediates T cell exhaustion and inhibits T cell immune response, which leads to tumor immunosuppression through TIM-3 specific binding (Ji et al., 2018). In the future, the potential combination of lncRNAs targeted drugs and tumor immune checkpoint inhibitor antibodies may be used for experimental treatment of human cancer. Considering that the six m^6^A/m^5^C/m^1^A-related lncRNAs were all protective factors of HNSCC (HR < 1), we analyzed the survival of the high-risk and low-risk subgroups of these 6 lncRNAs, and found that the prognosis of the high-expression subgroup of 5 lncRNAs was significantly better than that of the low-expression subgroup, which also proved their role as protective factors. The high expression of AL035587.1 and AC106820.5 is thought to correlate with the type of Immune C3 and C6 invasion, indicating their tumor suppressive effect. Estimatedscores (a combination of stromalscore and immunescore) showed that high expression levels of AC009121.3, AC106820.5 and FMR1-IT1 were significantly associated with lower tumor purity, while AL035587.1 and AF131215.5 had the opposite effect (Sawatani et al., 2020).

Drug sensitivity analysis showed that Paclitaxel, Docetaxel, and Gefitinib had higher sensitivity in the low-risk group than in the high-risk group, while Methotrexate had higher sensitivity in the high-risk group. These four drugs are commonly used in the clinical treatment of HNSCC (Yin et al., 2019; Ham et al., 2020; Guigay et al., 2021), and our results also demonstrate their value in the application of HNSCC. For the therapeutic potential of these 6 lncRNAs, we analyzed their sensitivity to different small molecule drugs. The results showed that PTOV1-AS2 was sensitive to four small molecular agents (Nelarabine, Decitabine, Acrichine, Dasatinib) (*p* < 0.05), and FMR1-IT1 was sensitive to five small molecular agents (Nelarabine, Chelerythrine, Tamoxifen, Tanespimycin, Sunitinib (*p* < 0.05). These eight drugs have certain anticancer activity and can be considered as candidates for the targeted therapy of HNSCC. Nelarabine is a purine analogue approved for the treatment of patients with T-cell lymphoblastic lymphoma (Teachey and O’Connor, 2020). Decitabine is a DNA demethylation agent, which plays an unmethylated role in inducing apoptosis and activating autophagy in myeloid leukemia (Li L. et al., 2021). Dasatinib is an antagonist of the SRC family of kinases, and may potentially induce T-cell response leading to improved survival of malignant pleural mesothelioma (Chen et al., 2020). Chelerythrine, Tamoxifen, Tanespimycin and Sunitinib have also been shown to have anticancer effects in a variety of cancers (Heng and Cheah, 2020; Wang and Liu, 2020; Ocadlikova et al., 2021; Su et al., 2021). These evidences also indicate the prospect of targeting these two lncRNAs for the treatment of HNSCC.

## 5. Conclusion

The poor prognosis of the HNSCC affects the health of tens of millions of people every year, and improving the prognosis of the HNSCC is particularly important. Studies have shown that m^6^A/m^5^C/m^1^A RNA methylation modifications was involved in the progression of cancer. Currently, many studies have chosen to use lncRNAs to establish prognostic markers, in order to provide new targets for diagnosis and treatment of cancers. Our study elucidates how m^6^A/m^5^C/m^1^A -related lncRNAs are related to the prognosis, immune microenvironment, and tumor mutation load of HNSCC. The prognostic signature can be used as an independent factor to better predict the prognosis of HNSCC, which may potentially become a new option for immunotherapy of HNSCC in the future.

## Data Availability

The datasets presented in this study can be found in online repositories. The names of the repository/repositories and accession number(s) can be found in the article/Supplementary Material.
