# Supplementary material for: The Prognostic Value and Immune Landscapes of a m6A/m5C/m1A-Related LncRNAs Signature in Head and Neck Squamous Cell Carcinoma
Source: Front Cell Dev Biol. 2021 Nov 30;9:718974. doi: 10.3389/fcell.2021.718974 (PMC8670092; doi:10.3389/fcell.2021.718974)
Supplement: Supplementary file 1 [file DataSheet1.zip › Supplementary materials/Supplementary figure1.docx]

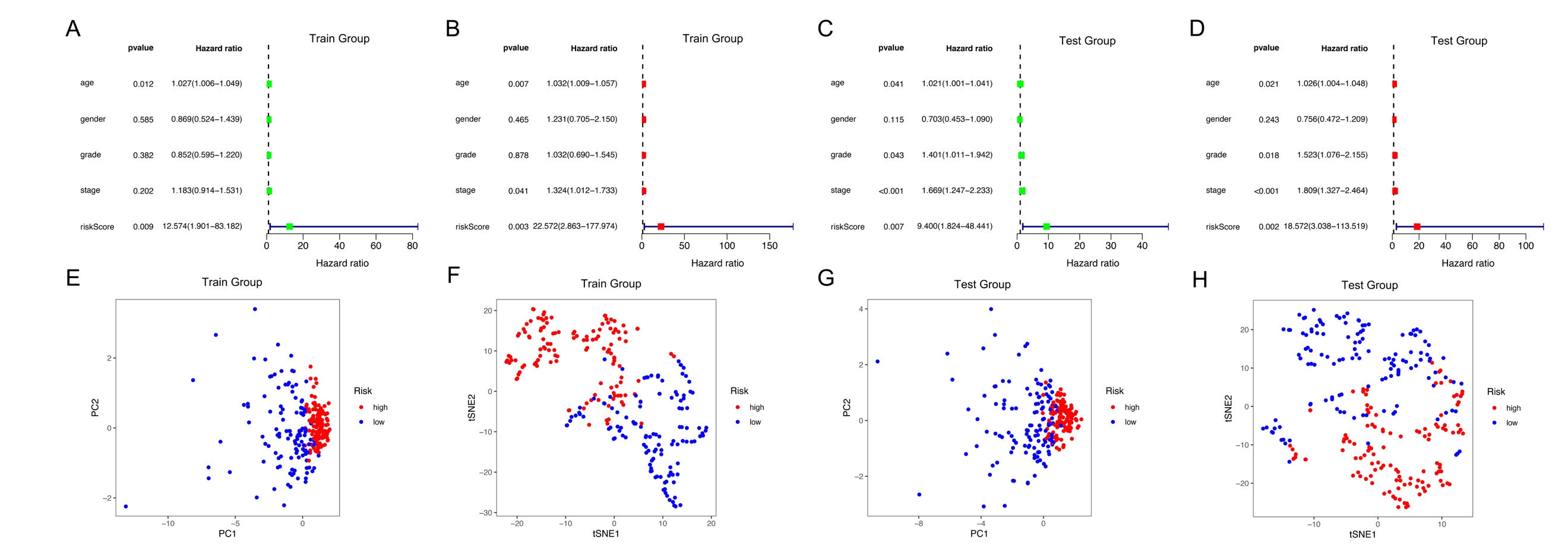


**(A-D)** The univariate and multivariate cox regression showed the predictive value of risk score compared with different clinicopathological characteristics in train group and test group. **(E-H)** Principal component analysis (PCA) and t-distributed stochastic neighbor embedding (t-SNE) of high-risk subgroup and low-risk subgroup in train group and test group.
