# Supplementary material for: The Prognostic Value and Immune Landscapes of a m6A/m5C/m1A-Related LncRNAs Signature in Head and Neck Squamous Cell Carcinoma
Source: Front Cell Dev Biol. 2021 Nov 30;9:718974. doi: 10.3389/fcell.2021.718974 (PMC8670092; doi:10.3389/fcell.2021.718974)
Supplement: Supplementary file 1 [file DataSheet1.zip › Supplementary materials/Supplementary Table1.docx]

**Supplement Table 1. Clinicopathological features of HNSCC patients in TCGA-HNSCC database.**

| **Covariates** | **Type** | **Total** | **Test** | **Train** | **P value** |
| --- | --- | --- | --- | --- | --- |
| age | <=65 | 324(65.06%) | 161(64.66%) | 163(65.46%) | 0.9251 |
| age | >65 | 174(34.94%) | 88(35.34%) | 86(34.54%) |  |
| gender | FEMALE | 133(26.71%) | 72(28.92%) | 61(24.5%) | 0.3111 |
| gender | MALE | 365(73.29%) | 177(71.08%) | 188(75.5%) |  |
| grade | G1-2 | 359(72.09%) | 183(73.49%) | 176(70.68%) | 0.8122 |
| grade | G3-4 | 120(24.1%) | 59(23.69%) | 61(24.5%) |  |
| grade | unknow | 19(3.82%) | 7(2.81%) | 12(4.82%) |  |
| stage | Stage I-II | 63(16.89%) | 29(15.34%) | 34(18.48%) | 0.5795 |
| stage | Stage III-IV | 301(80.7%) | 153(80.95%) | 148(80.43%) |  |
| stage | unknow | 9(2.41%) | 7(3.7%) | 2(1.09%) |  |
| T | T0 | 1(0.27%) | 1(0.53%) | 0(0%) | 0.1129 |
| T | T1-2 | 138(37%) | 61(32.28%) | 77(41.85%) |  |
| T | T3-4 | 233(62.47%) | 126(66.67%) | 107(58.15%) |  |
| T | unknow | 1(0.27%) | 1(0.53%) | 0(0%) |  |
| M | M0 | 184(36.95%) | 95(38.15%) | 89(35.74%) | 1 |
| M | M1 | 1(0.2%) | 1(0.4%) | 0(0%) |  |
| M | unknow | 313(62.85%) | 153(61.45%) | 160(64.26%) |  |
| N | N0 | 154(41.29%) | 87(46.03%) | 67(36.41%) | 0.0749 |
| N | N1-3 | 219(58.71%) | 102(53.97%) | 117(63.59%) |  |
| hpv-status | Negative | 72(14.46%) | 40(16.06%) | 32(12.85%) |  |
| hpv-status | Positive | 30(6.02%) | 13(5.22%) | 17(6.83%) | 0.3637 |
| hpv-status | unknow | 396(79.52%) | 196(78.71%) | 200(80.32%) |  |
