# Supplementary material for: The Prognostic Value and Immune Landscapes of a m6A/m5C/m1A-Related LncRNAs Signature in Head and Neck Squamous Cell Carcinoma
Source: Front Cell Dev Biol. 2021 Nov 30;9:718974. doi: 10.3389/fcell.2021.718974 (PMC8670092; doi:10.3389/fcell.2021.718974)
Supplement: Supplementary file 1 [file DataSheet1.zip › Supplementary materials/Supplementary figure3.docx]

**
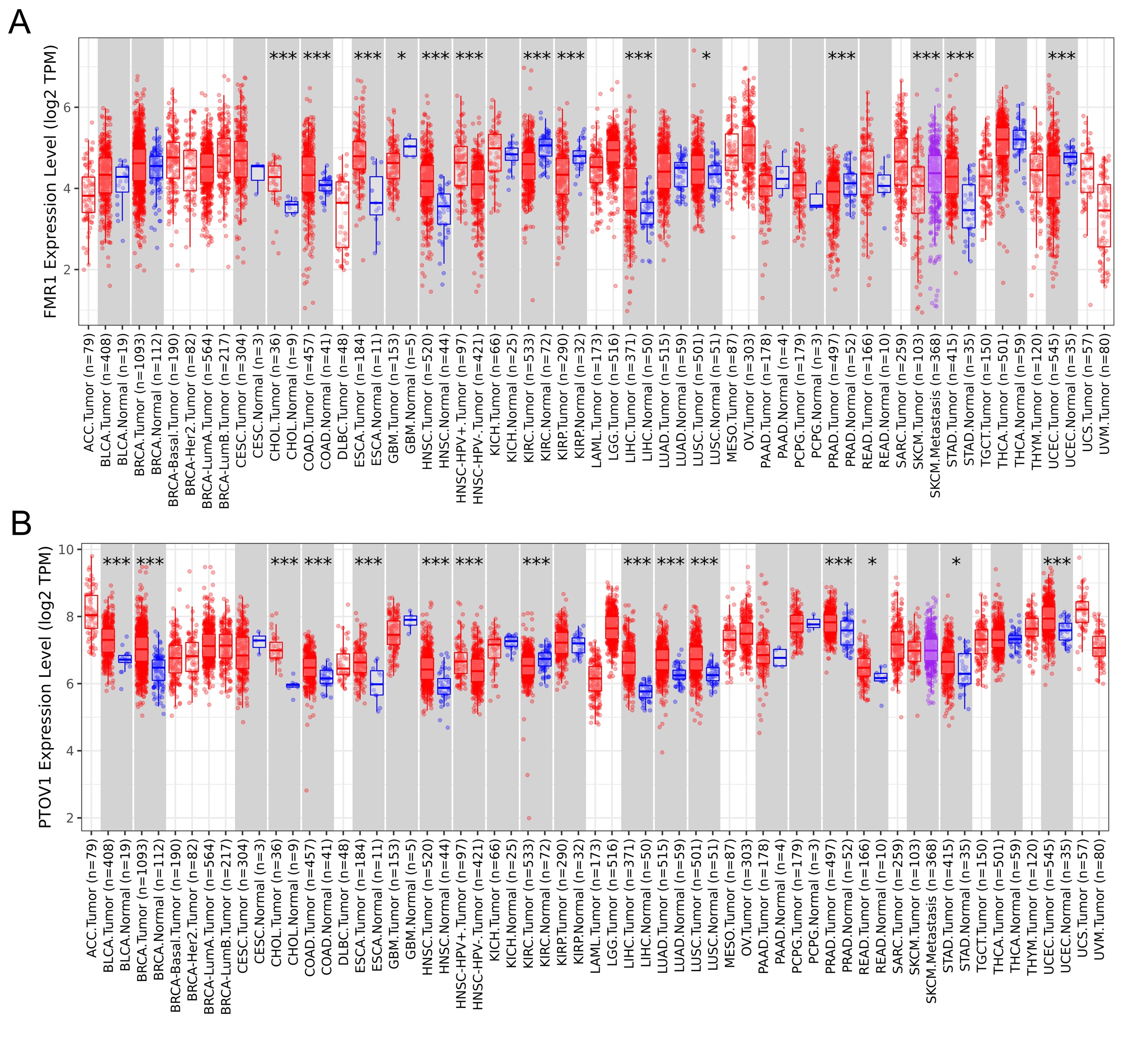
**

**Supplement Figure2. (A-B) The differential expression box plots of FMR1-IT1 and PTOV1-AS2 between tumor and adjacent normal tissues in TCGA HNSCC.**
