# Supplementary figures and images for: The Prognostic Value and Immune Landscapes of a m6A/m5C/m1A-Related LncRNAs Signature in Head and Neck Squamous Cell Carcinoma
Source: Front Cell Dev Biol. 2021 Nov 30;9:718974. doi: 10.3389/fcell.2021.718974 (PMC8670092; doi:10.3389/fcell.2021.718974)

**
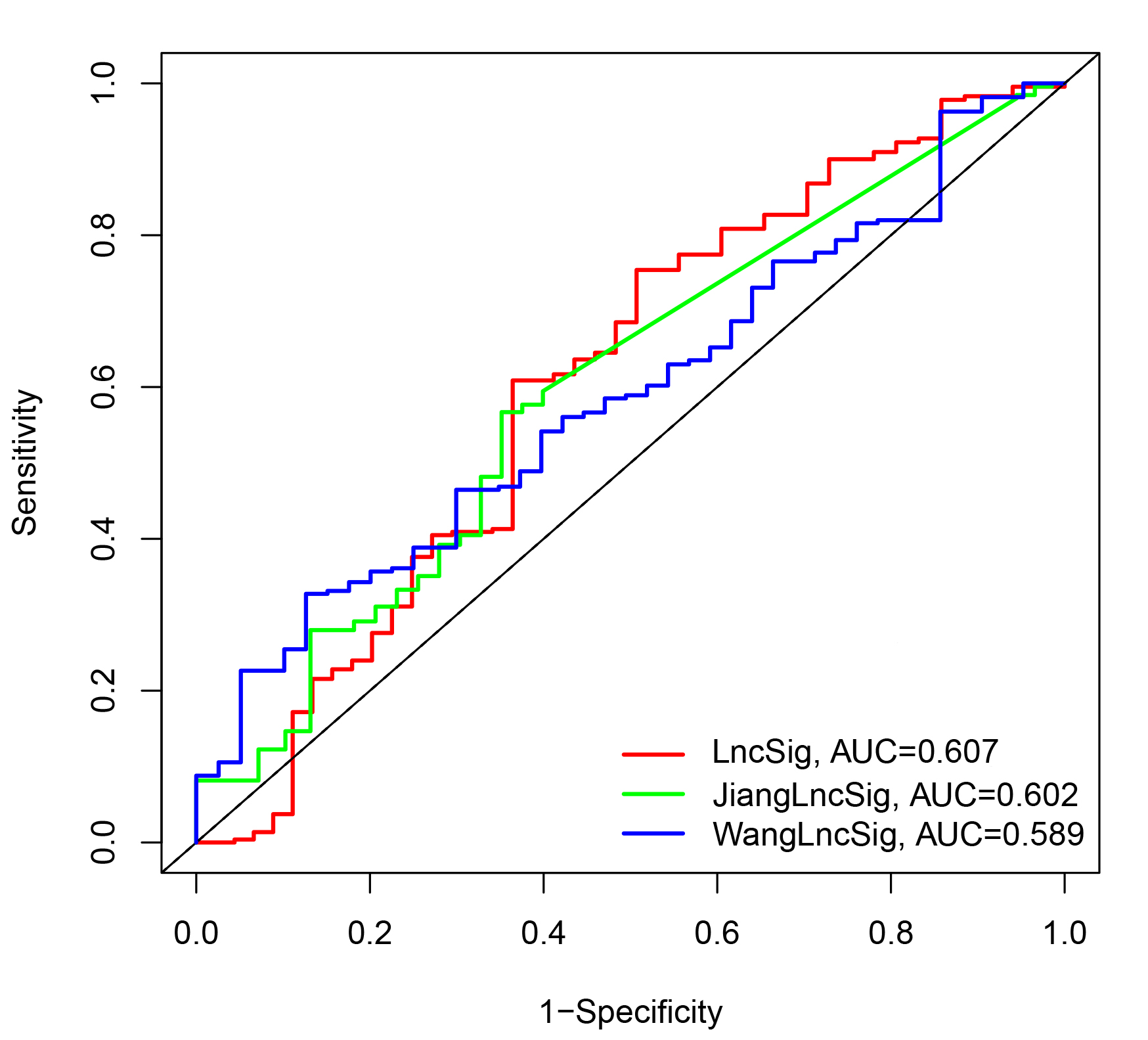
**

**Supplement Figure1. The ROC analysis at 5 years of OS for the LncSig, JianglncSig, Wang lncSig.**

Supplement: Supplementary file 1 [file DataSheet1.zip › Supplementary materials/Supplementary figure2.docx]

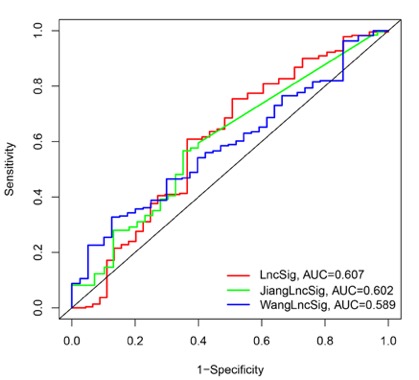

Supplement: Supplementary file 1 [file DataSheet1.zip › Supplementary materials/Supplementary figure2.jpg]
